# Estimating the Effective Size of European Wolf Populations

**DOI:** 10.1111/eva.70021

**Published:** 2024-10-22

**Authors:** Joachim Mergeay, Sander Smet, Sebastian Collet, Sabina Nowak, Ilka Reinhardt, Gesa Kluth, Maciej Szewczyk, Raquel Godinho, Carsten Nowak, Robert W. Mysłajek, Gregor Rolshausen

**Affiliations:** ^1^ Research Institute for Nature and Forest Geraardsbergen Belgium; ^2^ Ecology, Evolution and Biodiversity Conservation Leuven Belgium; ^3^ Senckenberg Research Institute and Natural History Museum Centre for Wildlife Genetics Gelnhausen Hessen Germany; ^4^ Department of Ecology, Institute of Functional Biology and Ecology, Faculty of Biology University of Warsaw, Biological and Chemical Research Centre Warszawa Poland; ^5^ LUPUS Institut für Wolfsmonitoring Und ‐Forschung in Deutschland Spreewitz Germany; ^6^ Department of Vertebrate Ecology and Zoology, Faculty of Biology University of Gdańsk Gdańsk Poland; ^7^ Centro de Investigação Em Biodiversidade e Recursos Genéticos, InBIO Laboratório Associado, Campus de Vairão Universidade Do Porto Vairão Portugal

**Keywords:** *Canis lupus*, conservation biology, conservation genetics, grey wolf, population genetics ‐ empirical, wildlife management

## Abstract

Molecular methods are routinely used to estimate the effective size of populations (*N*_e_). However, underlying model assumptions are frequently violated to an unknown extent. Although simulations can detect sources of bias and help to adjust sampling strategies and analyses methods, additional information from empirical data can also be used to calibrate methods and improve molecular *N*_e_ estimation methods. Here, we take advantage of long‐term genetic and ecological monitoring data of the grey wolf (*Canis lupus*) in Germany, and detailed population genetic studies in Poland, Spain and Portugal to improve *N*_e_ estimation strategies in this species, and species with similar life history traits. We first calculated *N*_e_ from average lifetime reproductive success and detailed census data from the German population, which served as a baseline to compare to molecular estimates based on linkage disequilibrium and sibship frequency. This yielded a robust *N*_e_/*N*_c_ estimation that we used to calibrate molecular estimates of German, Polish and Iberian wolf populations. The linkage disequilibrium method was strongly influenced by spatial genetic structure, much more than the sibship frequency method. When *N*_e_ was estimated in local neighbourhoods, both methods yielded comparable results. Estimates of the metapopulation effective size seemed to correspond generally well with the sum of the estimates of local neighbourhoods. Overall, we found that the number of packs is a good proxy of the effective population size. Using this as a rule of thumb, we evaluated for all European wolf populations the *N*_e_ 500 indicator and concluded that half of the European wolf populations do not yet fulfil this criterion.

## 1 Introduction

The effective population size is a key parameter in evolutionary biology and conservation biology, because it reflects the evolutionary potential and the risk of inbreeding, and therefore provides information on the conservation status of the population in question (Hoban et al. 2020). The effective size differs from the census size in natural populations because not every individual contributes with equal likelihood to the next generation. In its purest form, the effective size reflects variance in reproductive output relative to ideal conditions (Waples 2022). In ideal Wright‐Fisher populations, the variance in reproductive success (*V*_*k*_) equals the average reproductive success *k*, and the effective size *N*_e_ equals the census size *N*_c_, as defined by the number of potential parents. In reality, the variance in reproductive success is typically much larger, thereby leading to an effective size that is on average an order of magnitude smaller than the census size (Hoban et al. 2020). Detailed data on *N*_c_, reproductive success and the variance therein, however, are not available in most cases. Because of this, the effective size is typically estimated using molecular methods.

Although there are many methods to estimate *N*_e_ from molecular data, these methods are all depending on numerous assumptions, which are either rarely met or rarely taken into account fully, and as a corollary, each method shows a different aspect of the effective size (Spieth 1974; Ryman, Laikre, and Hössjer 2019; Nadachowska‐Brzyska, Konczal, and Babik 2022; Waples 2024).

The most used method to estimate *N*_e_ relies on estimating the amount of linkage disequilibrium (LD) among alleles at independent loci (Waples 2024). Since LD increases with decreasing effective size, LD can be used to estimate *N*_e_, assuming no other factors affect LD (Waples and Do 2008). However, it is well known that isolation by distance in continuous populations, which occurs when the population extent is clearly larger than the so‐called neighbourhood size NS (Wright 1946), yields downwardly biassed *N*_e_ estimates with LD‐based methods (Neel et al. 2013; Gilbert and Whitlock 2015). The neighbourhood expresses the spatial extent within which dispersal is not limiting and mating can be considered random. It can be thought of as the area of a circle with radius 2σ, with σ being the mean dispersal distance (the distance between the birthplace of the parents and the birthplace of the offspring). This area of a single neighbourhood is called the breeding window. For example, in wolves in Germany, the mean dispersal distance was estimated to be nearly 60 km (Reinhardt et al. 2019). As long as one considers individuals within a breeding window with a radius of 120 km, the sampled population of wolves within that area can be considered panmictic. As a consequence, the *N*_e_ estimated within that breeding window will only yield the *N*_e_ of that neighbourhood, and not of the total population (Neel et al. 2013). When individuals are sampled across multiple breeding windows, however, simulations indicate that linkage disequilibrium increases due to population structure, biassing the *N*_e_ estimate downwards (Neel et al. 2013). In practice, it is often unclear how much bias there is due to population structure and how we should adjust the sampling and analysis strategy to get reliable *N*_e_ estimates.

Another frequently used method estimates the likelihood of finding close kin in a random sample of the population, the sibship being estimated with molecular methods (Wang 2009). The more close kin there are in the sample, the fewer parents contributed to that sample, and by extrapolation to the population. This sibship frequency (SF) method also assumes a closed population, although as long as immigration is small relative to the total population size, the bias seems small. It does not strictly assume random mating within the population, but it is sensitive to non‐random sampling (Wang 2016).

When the populations are not isolated, however, the effective size estimated by both methods differs from the inbreeding effective size or the variance effective size, which estimate how much genetic drift the population experiences (Ryman, Laikre, and Hössjer 2019). In populations that are not closed to immigration, gene flow can compensate for local genetic drift to the point that (at migration‐drift equilibrium) its effective size approaches the effective size of the entire metapopulation (Spieth 1974; Ryman, Laikre, and Hössjer 2019). However, since neither of the above methods actually estimates the net loss of genetic diversity (the interplay between loss of variance due to finite population size and increase in variance due to gene flow), we rarely have reliable estimates of the inbreeding *N*_e_ (Waples 2022). Here, we are interested in estimating the local effective size of a subpopulation, without taking immigration from other subpopulations into account termed *N*_ex_ in Ryman, Laikre, and Hössjer (2019). From these subpopulation *N*_ex_ estimates, we can in principle approach the metapopulation effective size as $N_{\text{eMeta}}=\sum N_{\mathrm{ex}}/\left(1-F_{\mathrm{ST}}\right)$ (Spieth 1974, Ryman, Laikre, and Hössjer 2019) (under the assumption of migration‐drift equilibrium in an island model). As long as the genetic structure is weak (*F*_ST_ is small and gene flow exceeds one migrant per generation), the sum of the subpopulation *N*_ex_ is a reasonable approximation for the metapopulation *N*_e_.

In this study, we take advantage of detailed ecological and genetic monitoring data of the German wolf population to estimate key life history traits (generation time, average reproductive success, and variance in reproductive success) to calculate the effective size of a population of which the census size is extremely well known. This estimate of *N*_e_ and its relation to the census size *N*_c_ form a reliable basis for which to compare *N*_e_ estimates derived from molecular methods and to identify how sensitive different methods are to violations of model assumptions and sampling designs. Knowing the *N*_e_/*N*_c_ whilst having reliable *N*_c_ estimates allows us to also identify biases of *N*_e_ estimations when applying molecular methods to other wolf populations. Here, we showcase this for an Iberian and a Polish wolf dataset.

## 2 Material and Methods

### 2.1 Census Size

The census size of a wolf population is not a straightforward parameter by itself, as it is interpreted differently across the literature and countries. Here, we use the definition of Waples (2022), which defines the census size *N*_c_ as the number of potential parents. Wolf population sizes, however, are often expressed as the total number of individuals, which includes juveniles and subadults. This is because the different age groups are rarely discernible in the field or from genetically based capture–mark–recapture studies. We here term this *N*_tot_ for the sake of distinction from *N*_c_.

Wolves typically occur in family groups consisting of a single pair of breeders (parents), subadult individuals in their second calendar year (surviving offspring of the previous year), and young of the year. Litter size averages six pups, but can go up to ten, rarely more (Jȩdrzejewska et al. 1996; Nowak and Mysłajek 2016). As such, the size of a typical wolf pack at peak size consists of two breeders, a few yearlings (< 2 year old) and pups (< 1 year old). Occasionally, there are multiple breeders in a pack, which typically happens when the resident male is replaced and daughters of the former male are still pack members (Sidorovich and Rotenko 2019; Pacheco et al. 2024). Depending on the timing of mortality, birth, and dispersal, the size of a single pack can vary within a year from 3 to > 15 individuals, typically including no more than two adult individuals. The average pack size in the Central European population at the end of winter, at least in Poland, is five wolves (Nowak and Mysłajek 2016; Mysłajek et al. 2018).

Since the *N*_e_ calculated from life history traits is based on the variance in reproductive success, we only included reproductively mature individuals to calculate *N*_c_. In Germany, wolf monitoring reports the number of packs, pairs and territorial (adult) solitary individuals, allowing for an easy calculation of *N*_c_, even though it ignores solitary stray individuals.

#### 2.1.1 The Central European Wolf Population

The Central European wolf population was founded around 2000 AD from dispersing Baltic wolves that crossed the Vistula River and settled along the Polish–German border (Szewczyk et al. 2019; Jarausch et al. 2021). This population grew rapidly with an expansion in Germany from one pack in 2000 to 184 packs in 2022 (data: dbb‐wolf.de; Reinhardt et al. 2019) and with a similar increase in Poland (Nowak and Mysłajek 2016; Nowak et al. 2017). Genetically, this population is considered a distinct conservation unit from adjacent populations in the Carpathians, the Alpine arc or the Baltic region (Szewczyk et al. 2021). Gene flow between the Polish and the German parts of the population is currently limited due to a fence that hinders wild boars (and other large mammals) in their cross‐border dispersal, in an attempt to slow down the spread of African swine fever.

### 2.2 Wolf Monitoring in Germany

The DBBW (Dokumentations‐ und Beratungsstelle des Bundes zum Thema Wolf) hosts a compilation of wolf data collected in the German federal states and makes it accessible to the general public (https://www.dbb‐wolf.de/). The data used here cover the occupation of wolf territories in Germany from 2000 to 2020. Each monitoring year (from the 1 May until the 30 April the next year) wolf territories were evaluated, and the following data were recorded: the name and location of the territory, whether a territory was occupied by a solitary animal, a pair or a pack, the genetic identification code of each animal, and the number of pups born each year in every territory. Most of these data are based on genetic analyses, accompanied by camera trap data.

Individual wolves are continuously monitored and genotyped on the basis of various DNA sources, such as scat, hairs, or kill swabs at 13 autosomal microsatellite loci and two sex‐linked markers according to Jarausch et al. (2021). Genotype data are used to establish sibship relations and identify packs. We combined these data with the DBBW data to track lifetime reproductive success of individual female and male wolves between 2005 and 2020. A reproduction event was deemed successful if the offspring survived to adulthood and established its own territory. This excluded offspring emigrating to other countries. We assumed that wolves established a territory at the age of two, and tracked for how many years they held a particular territory. The longevity per individual was calculated as the number of territorial years plus two. Only individuals that could be considered deceased by 2020 were included. Mean *k* and variance *V*_*k*_ reproductive success were used to calculate the *N*_e_ of a cohort of *N*_c_ (adults) using $N_{e}=\frac{\bar{k}N_{c}-1}{\bar{k}-1+V_{k}/\bar{k}}$ (equation 1; Kimura and Crow 1963).

Census sizes were taken from https://www.dbb‐wolf.de/wolf‐occurrence/confirmed‐territories/map‐of‐territories, which provides per monitoring year the number of packs, pairs and solitary territorial wolves (Table 1). We assumed two adults per pack. In order to compare different estimates (*N*_c_ to *N*_tot_), we also estimated *N*_tot_ here by assuming an average late winter pack size of five individuals. We then get $N_{\mathrm{tot}}=5N_{\text{packs}}+2N_{\text{pairs}}+N_{\text{solitary}}$.

**TABLE 1:** The number of territorial wolves in Germany expressed as singles, pairs and packs between 2006 and 2022, the census size *N*_c_ (adults) and the total population size (*N*_tot_) deduced from that, assuming an average pack size of five wolves. Given the average and variance in reproductive success across that time period, we calculated the *N*_e_ on the basis of life history traits (*N*_e_ LH).

Year	Singles	Pairs	Packs	*N*_c_	*N*_tot_	*N*_e_ (LH)
2022	22	47	184	484	1036	186
2021	25	58	162	439	951	169
2020	22	37	159	414	891	159
2019	11	46	131	367	758	141
2018	12	42	105	306	621	118
2017	4	42	76	240	468	92
2016	3	23	60	171	349	66
2015	4	21	47	140	281	54
2014	6	19	32	108	204	42
2013	4	12	25	78	153	30
2012	3	12	17	61	112	23
2011	4	5	14	42	84	16
2010	6	7	7	34	55	13
2009	4	2	7	22	43	8
2008	4	3	5	20	35	8
2007	2	3	3	14	23	5
2006	1	0	3	7	16	3

### 2.3 Genetic Estimates of *N*_e_

#### 2.3.1 Germany

A total of circa 2500 unique wolf genotypes were present in the German database from 2000 to 2021. We split the data into three cohorts of 5 years each, containing genotypes present in 2006–2010, 2011–2015 and 2016–2020. We simulated 500 families based on the allele frequencies and calculated for four types of dyads (parent‐offspring, full‐sib, half‐sib and unrelated pairs) the distribution of coancestry coefficient values (Wang 2002) using the Related package (Pew et al. 2015) in R Statistical Software (v4.2.1; R Core Team 2022). A cutoff of 0.45 was selected to prune the dataset from local parent‐offspring or full‐sib relations. This pruning was done at three different home range sizes (diameters of 10, 15 and 25 km). This avoids the inclusion of close kin within the same territory (non‐dispersed offspring), which would otherwise inflate linkage disequilibrium among alleles and average sibship estimates used to calculate *N*_e_. When assuming a circular territory, the different pruning ranges correspond to territory sizes of roughly 80, 175 and 490 km^2^.

### 2.4 Overall Methodology Across all Populations

We estimated *N*_e_ for each unit on the basis of linkage disequilibrium (LD) using the software *N*_e_ estimator V.2 (Do et al. 2014). We also used the sibship frequency (SF) method of Jones and Wang (2010) in COLONY 2.0 to estimate the *N*_e_, using both options of random and non‐random mating. All individuals were considered as potential parents, with separate input files for males and females when sex was known. We used the full likelihood method implemented in COLONY 2.0. We assumed monogamy, which is the typical mode of reproduction, although new pairs are formed occasionally when a partner dies or is replaced by another wolf. When estimating *N*_e_. for the total population, we allowed for inbreeding due to population structure and assumed non‐random mating (estimated by the model itself on the basis of the data), given that there is known population structure across the Polish population (Szewczyk et al. 2019). For subpopulations, we assumed random mating within each subpopulation and no inbreeding due to population structure. Allelic dropout rate was set to 0.01 and genotyping error rates to 0.0005 across all loci. The probability of detecting a parent in our dataset was set to 0.9 for the German data, and to 0.27 for males and 0.17 for females in the Polish data, which were the frequencies detected by the model in the first iteration. For the Iberian data, we had no information on sexes, so the model was run with the 218 genotypes as offspring data.

Linkage disequilibrium‐based estimates of *N*_e_ included alleles of all frequencies for the German population, since we have a nearly perfect sample of the true population. These results were then compared to the life‐history‐based *N*_e_ estimation, in order to identify biases or sources of error.

We also applied *N*_e_ estimation methods on two other publicly available datasets: one from the entire Iberian wolf population (Silva et al. 2018; 218 genotypes at 48 microsatellite loci) and one from the entire Polish population (including Lithuania and N‐Slovakia; Szewczyk et al. 2019; 451 genotypes at 13 loci). For both populations, we also have estimates of *N*_c_ and/or *N*_tot_ (Boitani et al. 2022). Unlike in the German subpopulation (Jarausch et al. 2021), there is a known genetic and/or ecological structure in these populations, as described in the respective papers. Silva et al. (2018) consider the data as either consisting of four subpopulations or 11 subpopulations, for a total estimated population size of 2550 wolves belonging to an estimated 350 packs, 300 of which for Spain and 50 for Portugal (Boitani et al. 2022). Some individuals were not assigned to subpopulations at each hierarchical level and were excluded for some analyses, explaining the difference in total sample sizes across different analyses. Individuals identified as dispersers by Silva et al. (2018) in region A from region B were still analysed with region A. Szewczyk et al. (2019) considered 10 subpopulations across three management units (Central Lowlands, Baltic and Carpathian) for a total population size of circa 2400 wolves for Poland, Lithuania and N‐Slovakia combined. This census was based on genetic capture–mark–recapture models. Note that the average pack size across Europe is typically assumed to be eight (Boitani et al. 2022). However, average pack sizes are often closer to five wolves per pack (Liberg et al. 2015; Nowak and Mysłajek 2016; Mysłajek et al. 2018), but this can vary from three where hunting pressure is large (Okarma et al. 1998; Nakamura et al. 2021) to eight wolves per pack on average in some regions (Nowak et al. 2024). Converting *N*_tot_ into the number of packs (5–6 wolves/pack), we get an estimate of 400–480 packs, approximately.

We repurposed these datasets (archived in Godinho and Silva 2018; Szewczyk and Mysłajek 2019) for both *N*_e_ estimation methods either assuming them to be single panmictic units (to identify the scale of bias caused by population structure on *N*_e_ estimates), or in subdivided groups as reported in the respective papers. When reporting subpopulation *N*_e_ values and comparing them to the total dataset, we take the sum of the subpopulation *N*_e_ estimates. As long as *F*_ST_ is small (< 0.2), this hardly affects the metapopulation *N*_e_ (Ryman, Laikre, and Hössjer 2019). Both methods assume closed populations, but in the presence of gene flow, they provide the theoretical local inbreeding *N*_e_ in the absence of the mitigating effect that gene flow has on genetic drift (Ryman, Laikre, and Hössjer 2019), not the realised inbreeding *N*_e_. For *N*_e_ estimator, we used no cutoff of allele frequencies for the entire population, as these populations were sampled exhaustively or sample sizes were very large, and rare alleles in the entire sample are likely true representations of underlying patterns. When sampling subpopulations, we set the cutoff for rare alleles (which can be 0.05, 0.02 or 0.01) as the nearest value to the reciprocal of the sample size, in order to avoid a bias caused by rare alleles in the sample.

We used the full likelihood method implemented in COLONY 2.0. Marker‐type error rates were set to 0.01 and 0.0005 across all loci. The probability of detecting a parent was first calculated by identifying the frequency of parent‐offspring dyads.

We compared these estimates to expected estimates from the *N*_e_ */N*_c_ ratio calculated for the German population and used this to identify potential biases in the molecular methods, which may be linked to sampling design and method assumptions.

## 3 Results

### 3.1 *N*_e_ Estimates Across German Samples

**FIGURE 1:**
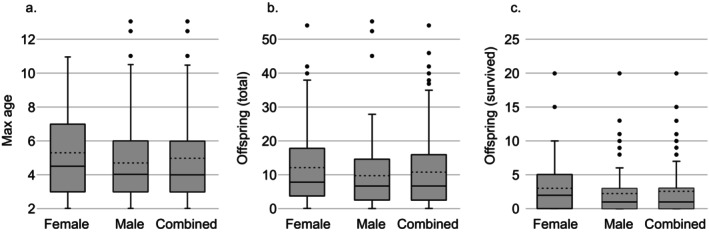
Summary of life history traits used to calculate *N*_e_/*N*_c_. (a) generation interval (max. age), (b) total reproductive investment (offspring total) and (c) realised reproductive output (offspring survived) for wolves in Germany, per sex and both sexes combined, represented as box plots. Median values: Full line; average values: Dashed lines. Whiskers represent 1.5 × the interquartile range.

Overall, within the period 2006–2020, we recorded 65 paired females and 90 males, born and likely deceased (not recorded afterwards as territorial individuals) within that period. In some territories, we observe a turnover period between the last recorded presence of a certain individual and the first presence of a new individual during which no genotype was recorded. In those cases, ½ year was added to the age. Assuming that individuals settled at the age of 2 years, adult males reached an average age of 4.7 years, whereas adult females lived on average until the age of 5.3 years (Figure 1). The overall generation interval is therefore considered 5.0 years, which we used to define three cohorts for the period 2006–2020 (2006–2010, 2011–2015, 2016–2020). Females and males that held a territory for at least 1 year produced across their lifetime between 0 and 54 pups (Figure 1). Females produced on average 12.4 pups (var: 144.0), of which 3.0 established a territory of their own (var: 14.9). Males produced on average 10.1 pups (var: 111.6) of which 2.28 established themselves (var: 12.1). Both sexes combined, we find an average reproductive success (the number of offspring surviving to adulthood) *k* = 2.59 (*V*_*k*_ = 13.4). From the difference between offspring production and survival to adulthood, we can deduce a mortality rate among immature individuals of circa. 50% per year.

Solving equation 1 for *N*_e_/*N*_c_ (only considering adult wolves for *N*_c_), we find the effective to census size ratio to be *N*_e_/*N*_c_ = 0.44 for females and 0.34 for males. Not taking sex‐specific differences in reproductive success into account, the overall *N*_e_/*N*_c_ is 0.38. Note that this *N*_c_ is not the total population size *N*_tot_ (which includes immature individuals and non‐territorial adults); it only represents the number of territorial wolves and thus potential parents. Translating this into the life‐history‐based effective size of the grey wolf population in Germany for consecutive years, we can input these ratios and compare them to the number of potential parents (within packs, pairs and territorial solitary individuals), as provided by the German authorities (DBBW) (Table 1). Genotypically based *N*_e_ estimates for the German wolf population are presented in Table 2 for the three cohorts and the three spatial sampling scales. In all three spatial sampling schemes, we find comparably low *N*_e_ point estimates for the first sampling period (2006–2010) across all three methods (range: 7–16), but the difference increases in the period 2011–2015 (range: 21–66) and period 2016–2020 (45–164), with LD consistently yielding the lowest values.

**TABLE 2:** Genetic estimates of *N*_e_ of the German wolf population, sampled as three cohorts between 2006 and 2020.

Radius	Cohort	SF rm	95% CI	SF nrm	95% CI	LD	95% CI
10	2006–2010	14	7–30	15	8–32	9.1	6.8–12.3
10	2011–2015	66	48–95	59	42–86	21.9	19.4–24.6
10	2016–2020	164	135–203	159	129–197	57.0	52.8–61.6
15	2006–2010	15	7–35	16	8–38	7.6	5.2–10.8
15	2011–2015	64	45–93	59	41–86	28.5	24.8–32.9
15	2016–2020	133	105–171	129	101–165	50.6	46.5–55
25	2006–2010	9	4–24	11	5–26	7.0	4.0–11.1
25	2011–2015	43	28–66	37	24–60	21.2	18.2–24.7
25	2016–2020	90	69–123	87	65–119	45.0	40.8–49.7

Abbreviations: 95 CI, 95% confidence intervals; LD, *N*_e_ based on linkage disequilibrium; Radius, Radius of the circle (km) representing a home range within which close kin was excluded from the dataset; SF, *N*_e_ based on sibship frequency (SF) with random mating (rm) or non‐random mating (nrm).

### 3.2 *N*_e_ Estimates of Polish and Iberian Wolf Populations

When we consider all samples of the Polish dataset (including Poland, Lithuania and N‐Slovakia) as panmictic, we get large differences between the SF‐based estimate (496) and the LD‐based value (63) (Table 3). Similarly, when we consider all samples within each management unit (Central Lowlands, Baltic and Carpathian), we also find lower LD *N*_e_ than SF *N*_e_ values (Table 3), likely reflecting a bias due to the deviation from random mating expected when different subpopulations are combined in the same analysis, leading to increased LD (Neel et al. 2013). When we analyse the 10 groups considered by Szewczyk et al. (2019) separately, we get point estimates between 17 and 64 per subpopulation. In this latter case, the correlation between SF‐ and LD‐based values of the subpopulations was moderately strong (Pearson *r* = 0.77). Metapopulation *N*_e_, estimated as the sum of subpopulations' *N*_e_, was much more similar for both statistics, 356 for SF *N*_e_ and 378 for LD *N*_e_.

**TABLE 3:** Molecular *N*_e_ estimates for the Polish+ dataset from Szewczyk et al. (2019).

Region	Unit	*N*	SF (rm)	95% CI	SF (nrm)	95% CI	LD	95% CI
TOT	PL + LT + SK	451^a^	496	430–571	420	359–493	63.5	59.6–67.7
CL	WPL I	81	49	34–73	50	32–77	50.8	39.7–66.9
CL	WPL II	36	31	18–54	26	15–50	38.7	26.8–61.9
CL	NWPL	44	38	25–63	35	22–58	43.3	22.7–37.7
CL	VRV	15	18	8–48	19	10–49	8.5	4.8–16.2
CL	Sum CL	176	136		130		141.3	
CL	CL pooled	176	195	156–241	179	141–230	44.6	39.9–49.9
BAL	CePL	16	17	9–39	19	10–43	10.2	6.7–16.1
BAL	NEPL I	41	32	19–57	30	18–56	30.5	23.1–41.8
BAL	NEPLII	39	36	23–62	36	22–62	63.8	43.2–110.3
BAL	LT	58	45	29–72	45	30–75	64.3	49.9–86.9
BAL	SEPL	23	27	15–56	26	14–56	16.7	11.6–25.6
BAL	Sum BAL	177	157		156		185.5	
BAL	BAL pooled	177	334	273–413	307	247–384	116.8	101.1–136.3
CAR	CAR	71	63	43–94	61	43–89	50.9	41.8–63.2
TOT	SUM subpops	424	356		347	216–615	377.7	
TOT	Sum MU	424	592		547	431–703	212.3	

Abbreviations: 95% CI, 95% confidence intervals; BAL, Baltic population; CAR, Carpathian population; CL, Central Lowlands population; LD, *N*_e_ based on linkage disequilibrium; *N*, Sample size; SF (rm), *N*_e_ based on sibship frequency (SF) with random mating.

^a^ Szewczyk et al. (2019) included 27 dispersers not assigned to the spatial groups or genetic clusters. This explains the difference in sample size for the total dataset (451) versus the sum of the subpopulations or management units (424).

For the Iberian population, we find similar trends (Table 4): LD‐based *N*_e_ values for pooled subpopulations are much lower than those obtained from SF. In both types of methods, when considering subpopulations at smaller spatial scales that still represent relevant ecological and evolutionary units, we observe an increase in the meta *N*_e_. Notably, the meta *N*_e_ calculated as sum of the subpopulation effective sizes is relatively similar across the three calculations. As explained above, LD‐based methods are very sensitive to spatial genetic structure. However, the SF method seems to be more robust to spatial structure, as mentioned by Wang (2016). For this method, the discrepancy between the *N*_e_ estimate from the pooled samples and the sum of the subpopulation *N*_e_ estimates is relatively small.

**TABLE 4:** Molecular *N*_e_ estimates for the Iberian dataset from Silva et al. (2018).

1 population	*N*	SF rm	95% CI	SF nrm	95% CI	LD	95% CI
Iberian Peninsula	218	250	201–308	176	141–226	75.5	72.8–78.1
4 subpops							
Asturias	55	68	46–101	49	31–76	24.3	22.7–26.0
Portugal	47	84	56–129	63	41–101	27.7	25.8–29.9
Castilla y León	57	57	37–88	41	25–64	28.5	26.4–30.8
Galicia	59	89	60–131	70	47–104	47.1	42.6–52.3
Sum		298		223		127.6	
11 subpops							
Alto Minho	13	20	10–54	14	7–39	32.6	22.0–57.9
W Trás‐os‐Montes	11	28	12–145	21	9–103	19.4	14.2–28.9
E Asturias	22	16	9–34	13	7–31	10.0	8.6–11.7
E Trás‐os‐Montes	17	44	24–116	35	18–97	26.1	20.8–34.0
SE Asturias	20	29	17–58	22	12–47	15.1	13.1–17.7
W Asturias	14	46	21–560	33	14–296	19.6	15.2–26.5
Castilla y León	14	36	18–122	25	11–91	17.9	13.6–24.8
S Douro	6	15	6–237	14	5‐INF	7.6	3.3–16.2
W Galicia	54	90	63–133	71	48–115	50.8	45.7–57.0
C Asturias	31	27	16–47	20	11–39	16.7	14.9–18.8
E Galicia	11	22	10–65	17	8–49	18.5	13.3–27.9
Sum		373		285		234.3	

*Note:* The LD estimate for the Alto Minho population with an LAF of 0.05 seemed an outlier (61, 95% CI 32.0–346.9), so we took the value for an LAF of 0.02. Five genotypes out of 2018 were not assigned to any of these groups at *k* = 11.

Abbreviations: 95% CI, 95% confidence intervals; LD, *N*_e_ based on linkage disequilibrium; *N*, Sample size; SF, *N*_e_ based on sibship frequency with and without random mating (rm and nrm, respectively).

## 4 Discussion

Estimating *N*_e_ with molecular methods has become rather easy with the development of single‐sample estimators (Wang 2016; Waples 2024). However, each method relies on a particular set of assumptions. When these are not met, estimates are likely to be biassed. Linkage disequilibrium increases not only as a result of inbreeding (non‐independence of inheritance of alleles across unlinked loci) but also due to non‐random mating caused by spatial structure (Neel et al. 2013). The sensitivity to such assumptions is not well known in natural situations and even more rarely tested with empirical data. Simulations can help to identify best practices in sampling strategies, but they remain simplified versions of reality, and they also rely on knowledge of life history trait distributions, such as a dispersal kernel and the true spatial distribution of individuals. By using methods that rely directly on life history traits, and which are independent of molecular methods, we were able to reliably estimate *N*_e_ for grey wolves in Germany, and extrapolate the information to other well‐studied populations in Europe. This helped to identify the sensitivity of certain sampling strategies to model assumptions of *N*_e_ estimation methods. We were also able to provide reliable estimates of the effective size of different wolf populations in Europe and to offer proxies for *N*_e_ in the absence of genetic data.

The wolf populations that we sampled were not always in the strictest sense of actual populations: while the Iberian population can be considered isolated, wolves in Germany are part of a larger Central European population that includes wolves in western Poland and satellites in neighbouring countries such as Austria, Belgium, the Czech Republic, Denmark and the Netherlands (Boitani et al. 2022). Wolves in Poland consist of three more or less distinct wolf populations (Central lowlands, Baltic, Carpathian) that experience gene flow but are still genetically distinct (Szewczyk et al. 2021). Using them as a single unit, however, served the purpose of illustrating what happens if we ignore model assumptions of panmixia, and to show how we can adapt our strategies to still end up with reliable estimates of the effective population size.

### 4.1 Germany

The German wolf population is one of the most intensively studied wolf populations worldwide. Since its re‐establishment in 2000, it has been the focus of in‐depth ecological and genetic monitoring, yielding near‐pedigree level information for a large proportion of the individuals (Jarausch et al. 2021), while providing estimates on dispersal (Reinhardt et al. 2019) and on important life‐history traits (age structure, reproductive effort and output). This information has led to important findings in relation to the effective size and how to estimate it reliably:

1. The linkage disequilibrium method was not very sensitive to differences in the exclusion strategy for close kin likely belonging to the same pack: estimates for all three periods were very similar when we considered a 10, 15 or 25 km radius for close kin exclusion. The SF method deviated considerably more, especially in the more recent sampling period.
2. Over time, the wolf population grew considerably across the study period. Meanwhile, also deviations from random mating can be assumed to have increased as the spatial distribution of wolves increased. We can indeed observe that before 2015 the spatial extent of the German wolf population hardly exceeded a breeding window (the spatial extent covered by a genetic neighbourhood; Wright 1946), which corresponds in this population to a circle with radius of approximately 120 km. After 2016, however, the spatial distribution of the population and the samples started to clearly exceed this extent due to the ongoing population expansion. This may explain why the LD and the SF *N*_e_ estimates did not strongly differ in the first two periods but showed a two‐ to threefold difference in the last period (Table 2).
3. Overall, the German wolf genetic dataset yielded the best approximation of the life‐history‐based effective size when we used the SF method and excluded close kin within each other's home range given by a circle with radius of 15 km instead of 10 or 25 km. Whether or not the model assumed random mating made little difference. The SF method has other limitations, however, which were not explored here in particular. Notably, sample size should be in the same order of magnitude of the true *N*_e_, which was the case here (Wang 2016).
4. Given that the life‐history‐based *N*_e_ estimates should provide the best possible baseline estimate of the true *N*_e_ (Waples 2022), we found values for the effective size that are reasonably approximated by the number of packs.

We pooled samples across years into three discrete 5‐year cohorts. However, these cohorts are biassed towards the most recent years of each 5‐year period, as there were simply more samples available due to the strong population growth (circa 20% per year). As a result, we can assume the molecular estimates tend to reflect mostly the end of each 5‐year period.

### 4.2 Polish and Iberian Populations

The census size of the wolf populations sampled by Szewczyk et al. (2019) was 1886 for Poland, 504 for Lithuania and 600 for Slovakia (Boitani et al. 2022). However, since only N‐Slovakia was actually sampled, for the sake of comparing *N*_c_ to *N*_e_ we consider half of the Slovak population, bringing the total census size to circa 2700 wolves. Assuming average pack sizes of 8, this translates into 337 packs. Average pack sizes are generally smaller, but not every wolf is part of a reproducing pack. If we convert *N*_tot_ for the German population into the number of packs, we need a conversion that includes pairs, solitary and stray individuals. Table 1 indicates an average conversion ratio between *N*_tot_ and *N*_packs_ of 6/1 on average for the period 2012–2022. Including stray individuals (the number of which is unknown) might increase this ratio further. So with 2700 wolves, the corresponding number of packs is likely in the range of 337 (*N*_tot_/*N*_packs_ = 8) to 450 (*N*_tot_/*N*_packs_ = 6).

With SF, we found the *N*_e_ estimated for the total dataset to be very similar to the sum of the subpopulation *N*_e_ values, and to be of the same magnitude as the estimated number of packs (total: 420 and 496 assuming non‐random versus random mating, pooled: 347 and 356, respectively). The sampled Baltic wolf population (eastern Poland + Lithuania) had approximately 1550 wolves or 195–258 packs (assuming *N*_tot_/*N*_packs_ = 8–6), and when the *N*_e_ of the Baltic population of eastern Poland and Lithuania was estimated separately with SF this yielded a value of 157 (the sum of five group point estimates) to 334 (point estimate for the pooled Baltic samples). For the Central lowlands, we find a value of 136 (sum of group values) to 195 (pooled samples) for an inferred number of packs of 62–110.

For the LD‐based *N*_e_, we find a very strong underestimate of the *N*_e_ when all samples are pooled, likely due to a strong violation of the model assumption of random mating and underlying population structure (Neel et al. 2013; Gilbert and Whitlock 2015). This was expected, given the known population structure in the data and the significant and strong genetic structure among subpopulations (Szewczyk et al. 2019). When we take the separate predefined 10 groups, however, the sum of their *N*_e_ estimates is very similar to the value obtained from SF data (LD *N*_e_ = 378, SF *N*_e_ = 356). This remains true when we compare values obtained for management units (Central Lowlands and Baltic). When we compare SF and LD‐based estimates of *N*_e_ for the pooled samples within those from management units, we have values that are three to four times lower for the LD‐based compared to the SF‐based estimates (Table 3). Overall, we have the most concordant results between the two methods (SF and LD) at the smallest spatial scales, at which assumptions of random mating (for LD) are more likely to be met. Note that the ten predefined groups of Szewczyk et al. (2019) are approximately the size of a breeding window each (45,000 km^2^) and that ten breeding windows cover approximately the entire surface of the study area (400,000 km^2^).

For the Iberian dataset, we find a similar trend: LD underestimated *N*_e_ when known population structure was not considered (75.5 vs. 234.3 for the pool of all samples and for the sum of *N*_e_ of subpopulations, respectively). Our value for the pool of all samples is only slightly higher than that provided by Sastre et al. (2011) for the Iberian wolf population using LD *N*_e_ methods. These authors suggested that the low *N*_e_ for the Iberian population would either represent an overestimation of the census size for this population or a very strong bottleneck effect. We show here that the *N*_e_ values offered by Sastre et al. (2011) were dramatically underestimated due to pooling samples from differentiated subpopulations while using linkage disequilibrium methods, which are highly sensitive to population structure (Gilbert and Whitlock 2015). However, when the LD *N*_e_ method is applied individually per subpopulation, it produces reasonably similar values to the sibship frequency methods. Sibship frequency‐based estimates produced a much weaker discrepancy; here the model does not assume random mating, only random sampling (Wang 2016). Depending on the sampling scale and the model assumption of random mating, we get total estimates of 176 (single population, non‐random mating) to 373 (sum of 11 subpopulations, random mating). The Iberian wolf population has been rather stable over the past two decades with a slight increase. The best estimates indicate that it consisted of 350 wolf packs at the time the samples were taken (Boitani et al. 2022). Notably, the number of packs seems to be a decent proxy of the effective population size also for this wolf population. With regard to the scale of sampling, however, we can see that the spatial scale of the population structure of the Iberian population discovered by Silva et al. (2018) was much smaller than that observed in the Polish and German populations (Reinhardt et al. 2019; Szewczyk et al. 2019), and that dispersal rates and distances were also much smaller. Applying the same breeding window size (circa 45,000 km^2^) to define the appropriate spatial sampling scale would bias local *N*_e_ estimates in Iberian wolves downwards due to mixture LD and by underestimating the number of breeding windows.

### 4.3 The Ratio Between *N*_e_ and *N*_c_ in Wolves

Across all datasets, we can make inferences on the *N*_e_ to *N*_c_ ratio. Traditionally, *N*_c_ is considered as the number of adults in the population, but for wolf monitoring, census sizes are typically reported as the total number of wolves that can be observed (*N*_tot_), which includes immature individuals. Depending on assumptions related to average pack size (5–8), which can moreover vary threefold across the year due to mortality and dispersal, we get *N*_e_/*N*_c_ values that vary from 0.38 (*N*_c_ = number of territorial adults excluding all immature wolves) to 0.12 (*N*_tot_ = all wolves and assuming average pack size = 8) with exactly the same source data.

Whichever approach is used, it is important to clearly distinguish between them. In birds, census sizes are typically reported as the number of breeding pairs times two (Keller et al. 2020). This does not take immature individuals into account, making the census size a straightforward metric to use. This may also explain why in general the reported *N*_e_/*N*_c_ ratio in birds is higher than in mammals (Hoban et al. 2020): the reason may not be ecological and related to differences in life history across these taxonomic groups but might partly be caused by scientific culture of reporting and by methodological constraints for monitoring.

### 4.4 Using the Number of Packs as a Proxy for *N*_e_

The Convention on Biological Diversity recognises the importance of genetic diversity and has adopted the threshold of *N*_e_ > 500 as representing populations capable of maintaining the evolutionary potential present at the given time. This is now integrated into headline indicator 4.A for the Global Biodiversity Framework (https://www.post‐2020indicators.org/metadata/headline/A‐4) (Hoban et al. 2023). Since it is impossible to monitor all species and their populations genetically, it is important to find reliable non‐genetic proxies to evaluate the status of populations. Our analyses indicate that in wolves, the number of packs is a useful proxy for the present‐day effective size. It seems sensible to use this conversion for easy communication with policy and stakeholders when conservation goals are concerned and when reliable genetic *N*_e_ estimates are not available.

It remains to be tested to what extent these findings can be extrapolated to other wolf populations worldwide, but if we compare the molecular estimates in the other two cases (Iberia, Poland) with the number of packs reported in these same countries, these findings seem to be rather robust. Also in the Dinaric‐Balkan region, this rule of thumb seems to hold: Šnjegota et al. (2021) estimated *N*_e_ in the Dinaric‐Balkan region at spatial scale that seems appropriate for LD‐based methods: for the population in Slovenia, Croatia and most of Bosnia‐Herzegovina, they report a combined *N*_e_ = 109 for a total of 157 packs (census data from Boitani et al. 2022, corrected for packs shared among countries). This falls within the *N*_e_/*N*_c_ range we found here for the Iberian and the Polish+ populations.

We can apply this rule of thumb (*N*_packs_ ≈ *N*_e_) and evaluate the status of the different wolf populations recognised by Boitani et al. (2022) across Europe, and consider populations that regularly exchange migrants as a single well‐connected metapopulation (Table 5), either as indicated by proven regular genetic connectivity or by clearly continuous spatial distributions. This again assumes an average pack size of eight wolves (Boitani et al. 2022). The results of this scrutiny (Table 5) indicate that half of the European wolf populations are still too small and/or insufficiently connected for the *N*_e_ > 500 criterion. Note that increasing connectivity across European wolf populations (Laikre et al. 2016) suggest > 5 detected migrants per generation would result in some subpopulations to be merged for the evaluation of this criterion. Since many subpopulations are still expanding, and since connectivity also increases with population size, we can expect some of these metapopulations to fuse together in the decades to come. As for now, we considered the Baltic and the Central European populations separately (Szewczyk et al. 2019, 2021).

**TABLE 5:** Inferred contemporary effective size of different metapopulations of wolves in Europe.

Metapopulation	Size (*N*_tot_)	Inferred *N*_e_
Iberian	2500	313
Alpine + Italian	4232	529
Dinaric + Balkan + Carpathian	9550	1194
Baltic	2490 (4490)	311 (561)
Central European	1850	231
Karelian	290 (750)	36 (94)
Scandinavia	550	55

*Note:* The names in the leftmost column represent the ten wolf populations currently recognised in Europe, grouped into seven metapopulations. *N*_tot_: Total estimated population size (midpoint estimates from Boitani et al. 2022). Inferred *N*_e_ gives the *N*_e_ deduced from the number of packs, assuming eight wolves per pack (if not independently estimated). The Baltic population estimate is given without and with Belarus (between brackets). The Karelian population estimate is given without and with wolves from Russian Karelia (between brackets).

## 5 Conclusions

Estimating the effective size of natural populations accurately (low bias, high precision) from population genetic data relies on a good understanding of assumptions. We consistently found that LD *N*_e_ estimations were biassed downwards when population structure was not taken into account, even when samples were all from recognised genetic clusters and genetic structure was weak. When taking population structure into account at the smallest spatial scale, we found a good concordance between estimates based on sibship frequency and based on linkage disequilibrium. As expected, the metapopulation *N*_e_ was well approximated by the sum of subpopulation *N*_e_ values. Specifically for wolves, the number of packs within a population seemed to be a decent approximation of the effective population size, across distinct wolf populations. In the absence of genetic monitoring data, the monitoring of pack numbers, or of the total number of wolves (combined with a conversion to number of packs) may be useful when evaluating conservation outputs and goals for wolves.

## Conflicts of Interest

Joachim Mergeay is an Editorial Board member of Evolutionary Applications and a co‐author of this article. To minimise bias, they were excluded from all editorial decision‐making related to the acceptance of this article for publication.

## Data Availability

Wolf genotype and life history data from Germany are available from Dryad 10.5061/dryad.d51c5b0c3. Wolf data from Iberia and Poland are available from Godinho and Silva (2018), and Szewczyk and Mysłajek (2019).
